# Structured Transformation of Unstructured Prostate MRI Reports Using Large Language Models

**DOI:** 10.3390/tomography11060069

**Published:** 2025-06-17

**Authors:** Luca Di Palma, Fatemeh Darvizeh, Marco Alì, Deborah Fazzini

**Affiliations:** CDI Centro Diagnostico Italiano, Saint Bon 20, 20147 Milan, Italy; luca.dipalma@cdi.it (L.D.P.); fatemeh.darvizeh@cdi.it (F.D.); deborah.fazzini@cdi.it (D.F.)

**Keywords:** LLM, MRI, information extraction, structured report, unstructured medical text

## Abstract

Objectives: to assess the ability of high-performing open-weight large language models (LLMs) in extracting key radiological features from prostate MRI reports. Methods: Five LLMs (Llama3.3, DeepSeek-R1-Llama3.3, Phi4, Gemma-2, and Qwen2.5-14B) were used to analyze free-text MRI reports retrieved from clinical practice. Each LLM processed reports three times using specialized prompts to extract (1) dimensions, (2) volume and PSA density, and (3) lesion characteristics. An experienced radiologist manually annotated the dataset, defining entities (Exam) and sub-entities (Lesion, Dimension). Feature- and physician-level performance were then assessed. Results: 250 MRI exams reported by 7 radiologists were analyzed by the LLMs. Feature-level performances showed that DeepSeek-R1-Llama3.3 exhibited the highest average score (98.6% ± 2.1%), followed by Phi4 (98.1% ± 2.2%), Llama3.3 (98.0% ± 3.0%), Qwen2.5 (97.5% ± 3.9%), and Gemma2 (96.0% ± 3.4%). All models excelled in extracting PSA density (100%) and volume (≥98.4%), while lesions’ extraction showed greater variability (88.4–94.0%). LLMs’ performance varied among radiologists: Physician B’s reports yielded the highest mean score (99.9% ± 0.2%), while Physician C’s resulted in the lowest (94.4% ± 2.3%). Conclusions: LLMs showed promising results in automated feature-extraction from radiology reports, with DeepSeek-R1-Llama3.3 achieving the highest overall score. These models can improve clinical workflows by structuring unstructured medical text. However, a preliminary analysis of reporting styles is necessary to identify potential challenges and optimize prompt design to better align with individual physician reporting styles. This approach can further enhance the robustness and adaptability of LLM-driven clinical data extraction.

## 1. Introduction

Structured reporting has been widely endorsed by leading radiological societies, such as the Radiology Society of North America (RSNA) and European Society of Radiology (ESR), to standardize reporting, enhance clinical communication, and enable data-driven research [1,2,3]. Despite these recommendations, many institutions continue to rely on unstructured, free-text radiology reports, which often impede the consistent extraction of clinically relevant data and limit the ability to build large-scale, queryable databases for both research and quality improvement purposes.

Efforts to promote structured report templates have met with partial success, partly due to the additional time and effort required for manual data entry into predefined fields [1]. As a result, large-scale retrospective analyses, epidemiological studies, and the development of advanced imaging biomarkers remain constrained by incomplete or inconsistently documented information.

Natural Language Processing (NLP) has long been a cornerstone in the effort to extract and analyze information from unstructured radiology reports. For example, Trivedi et al. [4] introduced an interactive NLP tool designed to identify incidental findings in radiology reports. In a study involving 15 physicians, the tool demonstrated significant improvements in model performance and a significant reduction in time spent reviewing. Banerjee et al. [5] obtained ClinicalBERT++ by fine-tuning the BERT model on 3 million radiology reports to improve the detection and follow-up of critical findings in radiology reports, achieving high performance in both internal and external validation datasets and offering a scalable solution for automated alert notifications and retrospective tracking of critical findings [6].

More recently, Large Language Models (LLMs) have emerged as a transformative tool in radiology, enhancing report readability, improving patient-provider communication, and streamlining non-interpretive tasks. Recent studies have demonstrated the ability of LLMs to simplify complex medical terminology in radiology reports, making them more accessible to patients. For instance, one study found that four LLMs (GPT-3.5, GPT-4, Bard, and Bing) significantly reduced the reading grade level of radiology report impressions across various imaging modalities when prompted to simplify the language [7]. Similarly, another study highlighted the efficacy of an LLM in improving the readability of foot and ankle radiology reports, achieving statistically significant improvements in Flesch Reading Ease and Flesch-Kincaid Grade Level scores [8]. Additionally, LLMs have shown promise in enhancing the clarity of knee and spine MRI reports, further underscoring their potential to bridge the gap between technical jargon and patient understanding [9]. Beyond simplifying reports, LLMs have been utilized to enhance MRI request forms and automate protocol suggestions, reducing the burden on radiologists while maintaining high accuracy [10]. Other authors have investigated the use of LLMs in improving radiology reporting accuracy. A study by Gertz et al. evaluated GPT-4’s ability to detect common errors in radiology reports, such as omissions, insertions, and spelling mistakes, achieving an error detection rate of 82.7%, which was comparable to the performance of radiologists across all experience levels [11].

Despite these advancements, challenges remain in using LLMs to extract structured data from unstructured reports, particularly in specialized clinical contexts [12]. For instance, a study evaluating ChatGPT’s performance in determining LI-RADS scores based on MRI reports revealed poor accuracy (53% for unstructured reports and 44% for structured reports) [13].

Given these challenges, our study focuses on addressing a critical gap in the application of LLMs to radiology reporting: the extraction of structured data from unstructured MRI reports. While applicable to various radiological exams, we focused on multiparametric prostate MRI due to its clinical complexity and relevance to prostate cancer biomarkers. Thus, the aim of this study was to develop and validate an automated pipeline using open-weight LLMs and rule-based parsing to convert free-text prostate MRI reports into standardized, queryable formats. This approach enhances data utilization for research, quality improvement, and patient care, addressing a critical need in clinical workflows.

## 2. Materials and Methods

### 2.1. Data Acquisition, Cleaning, and Categorization

In this retrospective study, we used a set of unstructured, clinically generated, Italian-language prostate MRI reports. The ethic approval was obtained on September 11, 2024, by CET Lombardia 3 Ethical Committee (Study ID: 5105). One expert data scientist (L.D.P., with 2 years of experience working with LLMs) revised the reports in order to manage the dataset. Only minor changes were made; for example, many radiologists had appended a fixed legend at the end of the report to explain the meaning of each PI-RADS value. These common strings were removed before processing the reports with the LLM. The dataset was cleaned in a balanced manner across multiple radiologists. All medical reports used were standalone text reports that did not contain any personally identifiable information. Specifically, the reports included no names, patient IDs, dates of birth, or other direct or indirect identifiers. As such, the dataset was fully de-identified before any processing. To further protect data privacy, all processing was performed locally within the institution’s secure computing environment. No data were transmitted to external services or cloud-based platforms at any point during the study, thereby eliminating the risk of external exposure.

### 2.2. Feature Extraction by Radiologist

One radiologist performed manual extractions of key information, according to a list of predefined features referring to 3 entities: Exam, the main entity containing the sub-entities, Lesion, and Dimension.

The Exam entity is composed of:

- Volume (float)
- PSA density (float)
- Lesions (List of Lesion elements)
- Dimensions (Dimension element)

Where each Lesion is composed of:

- PI-RADS score (int)
- Location (str): allowed values are “Transition zone”, “Peripheral zone”, “Transition zone and peripheral zone”

While Dimension is composed of:

- Longitudinal dimension (float)
- Transverse dimension (float)
- Antero-posterior dimension (float)
- Unit of measurements (str): allowed values are “mm” and “cm”

These manually curated data served as the ground truth for subsequent model evaluation. Examples of reports and filled templates are presented in Table 1 and Table 2.

### 2.3. Model Validation

#### 2.3.1. Architecture

A diverse set of high-performing open-weight LLMs was selected to process unstructured medical text. The models varied in architecture and parameter size, allowing a comprehensive comparison. Specifically, we included:

- Llama3.3 70B parameters (Meta, Menlo Park, CA, USA), known for strong language understanding, performing similarly to Llama 3.1 405B [14].
- DeepSeek-R1-Llama3.3 70B parameters (DeepSeek, Hangzhou, Cina), a distilled version of DeepSeek-R1 on Llama3.3, demonstrating performance comparable to OpenAI models [15].
- Phi4 14B parameters (Microsoft, Redmond, WA, USA), a state-of-the-art open model balancing efficiency and performance [16].
- Gemma-2 27B parameters (Alphabet, Mountain View, CA, USA), excelling in multi-turn conversations and reasoning [17].
- Qwen2.5-14B 14B parameters (Alibaba Cloud, Hangzhou, China), offering specialized models and broad language support [18,19].

All models were tested with uniform inference settings: zero temperature (deterministic outputs) and unlimited token length (avoiding truncation), ensuring fair assessment of their clinical text interpretation capabilities.

#### 2.3.2. Prompting

**Figure 1:**
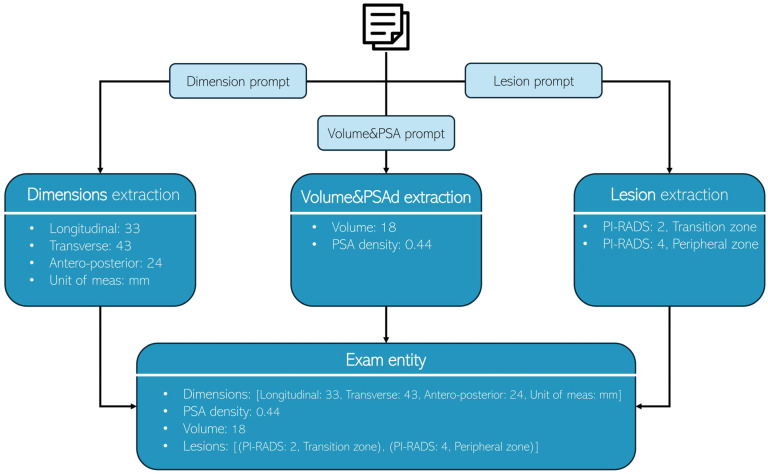
Each radiology report undergoes processing three times, with each iteration utilizing a distinct prompt to extract specific information: Dimensions, Volume & PSA, and lesion characteristics, respectively.

A structured three-step zero-shot prompting technique was adopted for all models to extract relevant information systematically (Figure 1). While each LLM utilized its own prompt format, a consistent system-prompt and user-prompt structure was applied to ensure fair comparison. This standardized approach preserved the unique prompt designs of individual models while maintaining an equitable evaluation framework. The extraction process was divided into three sequential steps:

- Dimension extraction: the first prompt focused on identifying and extracting prostate dimensions (longitudinal, transverse, antero-posterior, and measurement unit) from the provided medical report.
- Volume and PSA density extraction: the second prompt extracted the prostate volume and PSA density.
- Lesion extraction: the third prompt identified and extracted lesion details (PI-RADS and location).

The prompts used were:

- System Prompt:
  *“You are a highly skilled radiologist with extensive experience in prostate imaging and diagnostics, as well as proficiency in Python programming. Your task is to analyze the provided medical report and generate a comprehensive, professional radiology report following the provided instructions.”*
- User Prompt for dimension extraction:

*“Here is the report that you have to process:*

*[START REPORT]*

*- report here -*

*[END REPORT]*

*Instructions:*

- *Extract the dimensions mentioned in the report*.
- *Assign each dimension to its corresponding category: Anteroposterior (AP), Latero-lateral (LL), or Craniocaudal (CC, or Longitudinal)*.
- *If the report does not explicitly specify which dimension corresponds to AP, LL, or CC, or if the dimension names are not clearly stated, set dimensionOrder to: [NA, NA, NA]*.
- *Do not infer or assume dimension assignments based on context; rely solely on explicit information provided in the report*.

*Answer:*

*{{“dimensions”: Optional[List[float]],*

*“dimensionsOrder”: Optional[List[str]],*

*“measurement_unit”: Optional[Literal[“mm”, “cm”]]}}”*

- User Prompt for volume and PSA density extraction:

*“Here is the report that you have to process:*

*[START REPORT]*

*- report here -*

*[END REPORT]*

*Instructions:*

- *If the report explicitly specifies the prostate volume* (e.g., *“volume: 35 mL” or “volume: 35 cc”), extract that value and assign it to the ‘volume’ field*.
- *If the report provides only linear dimensions* (e.g., *“AAxBBxCC mm”) without explicitly stating the volume, leave the ‘volume’ field empty*.
- *If the report explicitly specifies PSA density* (e.g., *“PSA density: 0.15 ng/mL/cm^3^”), extract that value and assign it to the ‘psa_density’ field*.
- *If the report does not explicitly specify PSA density, leave the ‘psa_density’ field empty*.
- *Do not infer or calculate volume or PSA density based on provided dimensions or other context; rely solely on explicit information stated in the report*.

*Answer:*

*{{‘volume’: Optional[float], ‘psa_density’: Optional[float]}}”*

- User Prompt for lesion extraction:

*“Here is the report that you have to process:*

*[START REPORT]*

*- report here -*

*[END REPORT]*

*Instructions:*

- *Extract the lesions mentioned in the report*.
- *Assign to each lesion its corresponding pirads and location (peripheral zone, transition zone, peripheral and transition zone)*.

*Answer:*

*{{‘lesions’: Optional[list[pydanticLesion]]}},*

*where pydanticLesion is defined as:*

*{{‘pirads’: Optional[int], ‘location’: Optional[Literal[“Transition zone”, “Peripheral zone”, “Peripheral and transition zone”]]}}”*

The complete prompt configurations used for each model can be found in Table 3.

#### 2.3.3. Structured Output Schema

The same structured schema, mirroring the manually curated data, was utilized by the LLM to ensure consistency in feature extraction. The schema adheres to the predefined entities and their respective attributes, such that the LLM-generated data aligns with the ground truth established by the radiologist, facilitating accurate model evaluation.

### 2.4. Evaluation Metrics

The performances of each LLM were assessed using distinct evaluation matrices based on the extracted features:

- For longitudinal, transverse, antero-posterior, unit of measurements, volume, PSA density:
  ○ Accuracy: the proportion of correctly extracted entities compared to manual annotations. A prediction was considered correct when:
    ▪ both prediction and ground truth were empty or
    ▪ prediction and ground truth were equal.
- For lesions (example is shown in Table 4):
  ○ F1-Score: a more granular evaluation was performed to account for partial matches in lesion PI-RADS and location. The process involved calculating true positives (TP), false positives (FP), and false negatives (FN) as follows:
    1. For each examination, a blank set of new lesions was created based on the lesion ground truth.
    2. Each prediction was matched with the ground truth:
      ○ Complete match: predictions were first matched to the blank schema where both the PI-RADS score and lesion location matched the ground truth.
      ○ Partial match: remaining predictions were matched if at least one field (PI-RADS or location) matched the ground truth, reflecting cases where the model identified the correct lesion but made an error in one of the two fields.
    3. For the remaining predictions, any unmatched ones were used to fill blank spaces, with excess predictions classified as FP.
    4. For each lesion:
      - If there was a match in lesion PI-RADS: TP was incremented by 0.5.
      - If there was a match in lesion location: TP was incremented by 0.5.
      - If there was no match in lesion PI-RADS: FN was incremented by 0.5, and FP was incremented by 0.5.
      - If there was no match in lesion location: FN was incremented by 0.5, and FP was incremented by 0.5.
    5. Final exam score: The F1-score for each examination was computed using the formula:
      $$F1=\frac{2\;*\;TP}{2\;*\;TP+FN+FP}$$
- Processing time: the time required to process all reports. During the construction of the ground truth dataset, the time required for manual data extraction from scratch and the time for manual review of pre-filled templates were evaluated using a sample of examinations.

## 3. Results

### 3.1. Data Acquisition

**Figure 2:**
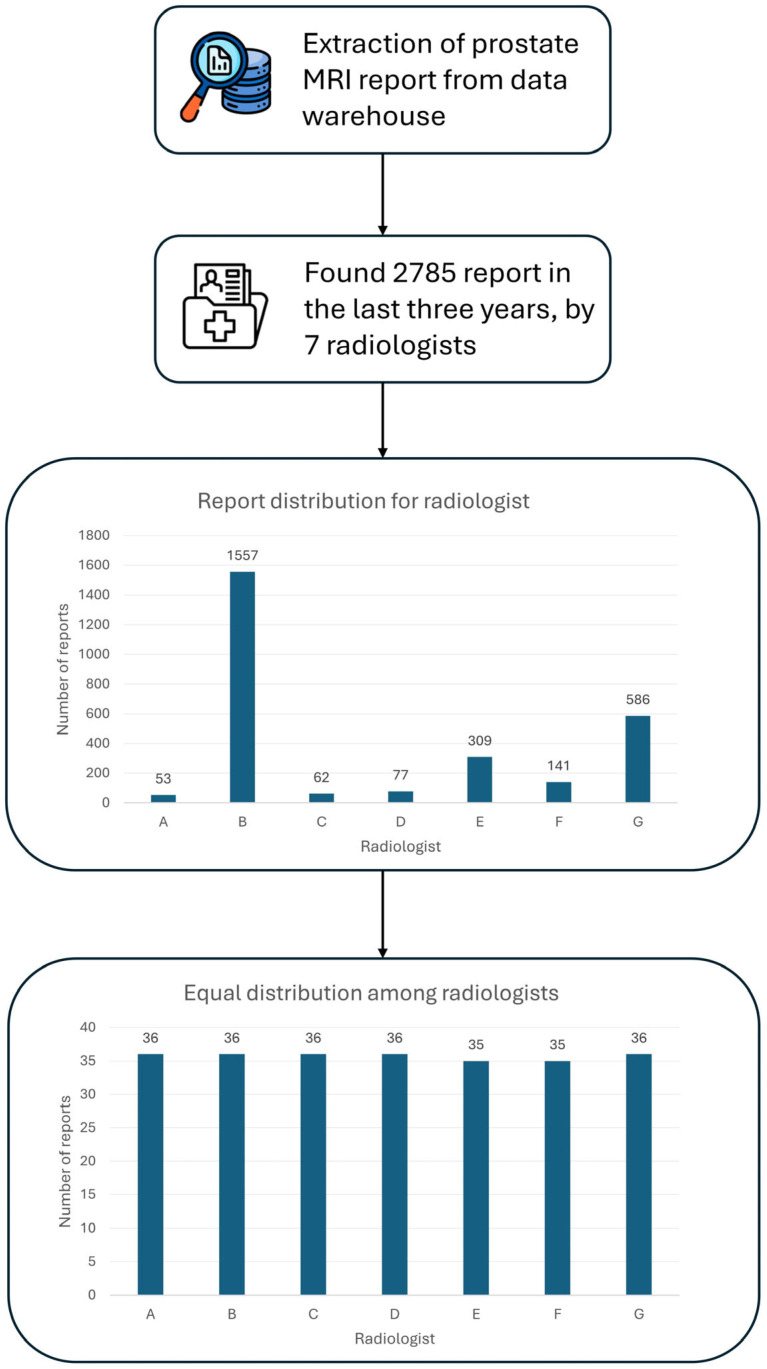
Workflow for dataset generation. The complete workflow illustrates the steps taken to obtain the final dataset of 250 radiology reports.

A total of 250 unstructured prostate MRI reports were retrospectively analyzed in this study. These reports were derived from routine clinical practice at our Center, comprising prostate MRI exams conducted over the last three years, amounting to 2785 exams (Figure 2). The minimum number of reports required per radiologist to reach a total of 250 reports and to ensure balanced representation among radiologists was determined. Seven radiologists with at least three years of experience in prostate MRI interpretation were selected. The study population comprised 248 people, ranging in age from 29 to 87 (66 ± 9), with a median prostate volume of 51.5 cc (IQR 37.5–73.5). Regarding anatomical prostate measurements, the median longitudinal dimension was 50 mm (IQR: 43–57 mm), while the transverse dimension measured 53 mm (IQR: 47–58 mm). The median antero-posterior dimension was recorded as 39 mm (IQR: 34–45 mm). However, these measurements were unavailable for 71 cases, corresponding to 28.4% of the total dataset. This missing data resulted from radiology reports in which prostate dimensions were recorded in the format ‘AAxBBxCC’ without specifying which value corresponded to the longitudinal, transverse, or antero-posterior dimension. As it was not possible to accurately determine the correct dimension assignment, these cases were considered missing in the analysis. The median PSA density was 0.09 ng/mL^2^ (IQR: 0.06–0.14), although data on PSA density were missing (the value was not specified by the radiologist in the original report) for 152 examinations, representing a significant portion (61%) of the cohort (Table 5A).

Analysis of lesion frequency per examination revealed that 20 scans (8%) showed no detectable lesions, while the majority of examinations (*n* = 130, 52%) identified a single lesion. Two lesions were detected in 70 examinations (28%), while 20 scans (8%) revealed three lesions, and 9 scans (3.6%) displayed four lesions. In one examination (0.4%), there was no clear definition of lesions and it was excluded from the count.

A total of 366 lesions were identified across all examinations. Among these, 237 (64.8%) lesions were classified as PI-RADS 2, while 53 (14.5%) were classified as PI-RADS 3. 58 (15.8%) lesions were found to be PI-RADS 4, and a smaller subset of 13 (3.6%) lesions were assigned a PI-RADS 5 classification. The remaining five (1.3%) didn’t have a clearly specified PI-RADS classification. Additional clinical characteristics, including PI-RADS score distribution and lesion localization, are detailed in Table 5B.

### 3.2. LLMs Output

The LLMs were running locally on our server using the Ollama (version (v) 0.4.6) framework. The output was obtained and validated using Instructor (v. 1.7.2) and Pydantic (2.9.2). Each report was processed 3 times, once for each task with its prompt to extract the different features (dimensions, volume & PSA density, lesions). The outputs of each task were then processed and used to fill the Exam entity. Examples of the expected outputs of each task are shown in Table 6.

### 3.3. Evaluation Metrics

**Figure 3:**
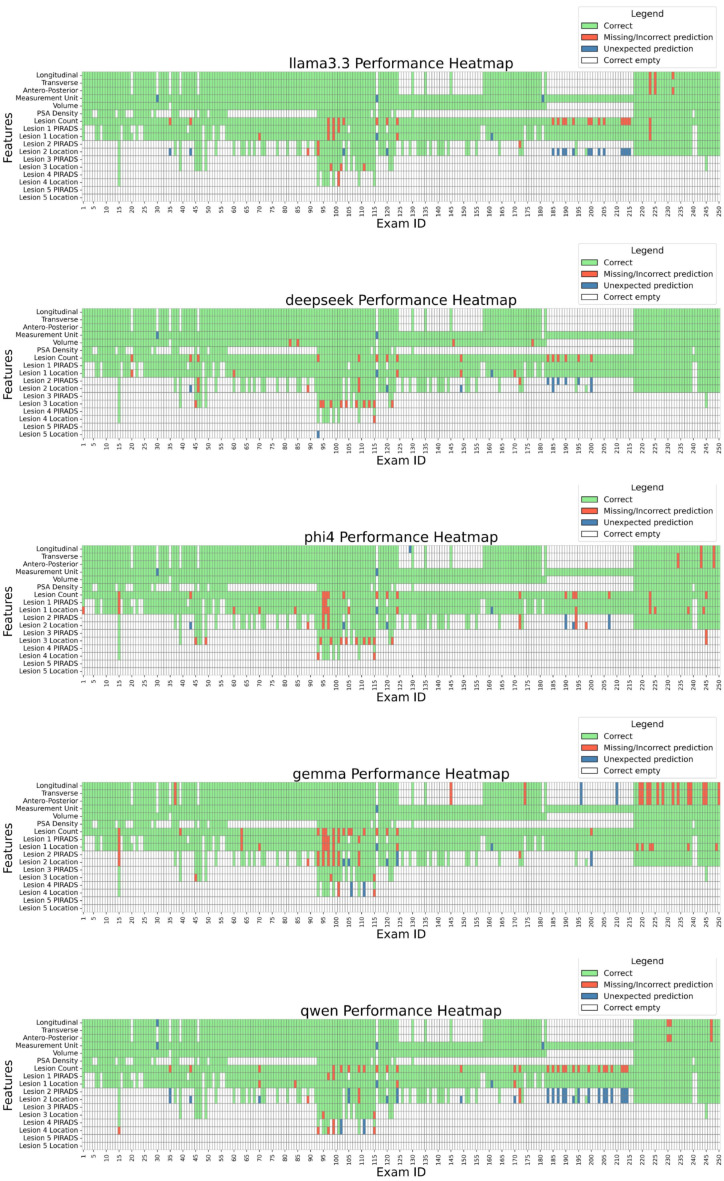
Model performance by exam and feature. The heatmap displays the performance of each model across individual exams and extracted features. LEGEND: Correct: the prediction matches the ground truth; Missing/Incorrect prediction: the prediction is missing or is different from the ground truth; Unexpected prediction: while the ground truth is missing, the LLM still made a prediction (hallucination). Correct empty: the ground truth was empty, and the LLM correctly left the field empty.

**Figure 4:**
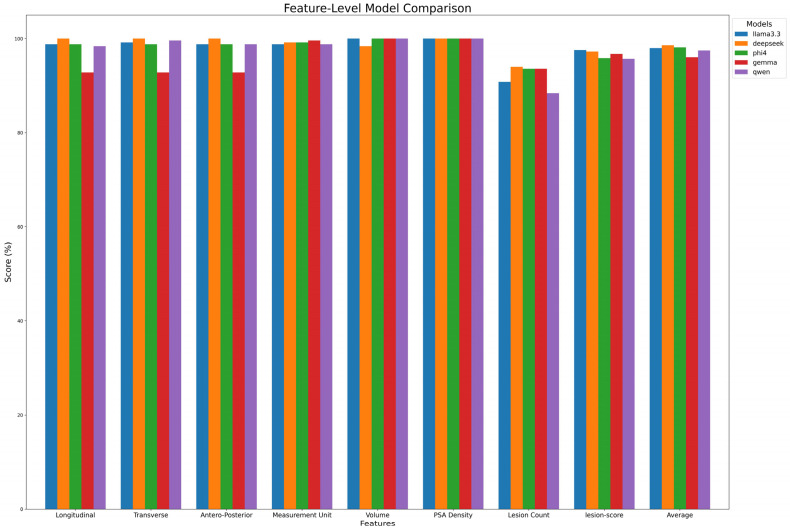
Model performance on feature extraction. The figure compares the performance of each model in extracting key features from radiology reports.

To assess open-source LLMs in extracting values from medical reports, we established a ground truth based on annotations made by Radiologist C. Models’ performances were evaluated by comparing their predictions with this ground truth. Figure 3 details performance case by case, while Table 7A and Figure 4 summarize extraction results across features. DeepSeek-R1-Llama3.3 achieved the highest average score (98.6% ± 1.97%), followed by Phi-4, Llama 3.3, Qwen, and Gemma. While models performed well in extracting Volume and PSA Density (~100%), variability was observed in lesion count extraction, with Qwen scoring the lowest (88.4%). Lesion-score extraction varied by model, ranging from Qwen2.5 (95.7%) to Llama3.3 (97.3%).

A qualitative analysis of model errors revealed several common failure modes. For example, some LLMs attempted to infer prostate dimensions (longitudinal, transverse, antero-posterior) even when the associations between the numbers and the dimensions were not explicitly specified in the report, leading to incorrect extractions. In other cases, units of measurement were incorrectly assigned, e.g., by mistakenly associating values with units mentioned elsewhere in the report rather than those relevant to prostate dimensions. Lesion-related errors included missing lesions entirely, misclassifying lesion location when the description was ambiguous, or failing to recognize that two references in the report referred to the same lesion, resulting in overestimation of lesion count.

**Figure 5:**
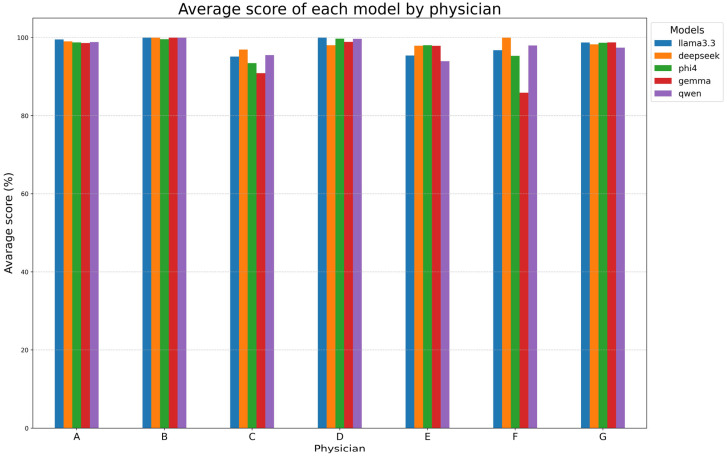
Model performance at physician-level. This figure evaluates the performance of each model with respect to each physician (A–G).

Table 7B and Figure 5 examine the performances of models for each reporting physician, with DeepSeek-R1-Llama3.3 again achieving the highest mean score (98.6% ± 1.1%). Llama 3.3, Phi-4, and Qwen2.5 followed closely, while Gemma-2 had the lowest mean score (95.8% ± 5.5%). Models performed exceptionally well on reports made by radiologists A, B, D, and G (~99%) but showed lower alignment with radiologists C, E, and F. Gemma-2 exhibited the most variability, while DeepSeek-R1-Llama3.3 maintained high consistency.

**Figure 6:**
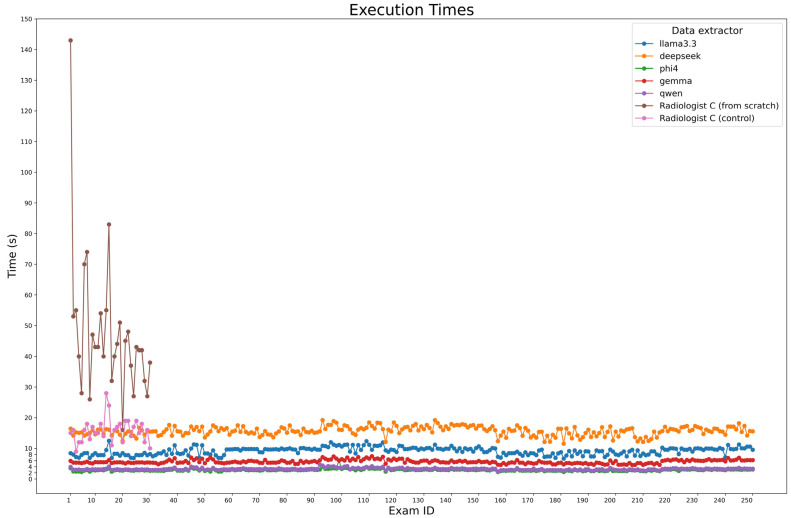
Execution time comparison. The plot compares the execution times of each model for processing all 250 cases, alongside the time.

During ground truth evaluation, Radiologist C was observed while compiling a sample of 30 examinations from scratch, achieving a median time of 43 s per report (IQR: 37.25–52.5). The same reports were used for a second examination, where radiologist C didn’t fill the fields from scratch but only corrected the model predictions, in particular DeepSeek-R1-Llama3.3 predictions, obtaining a median time of 16s (IQR: 13.25–18). For automated extraction, the execution times of the model were measured across all 250 cases using Python (v. 3.10.12) timing functions, considering all three steps made for entity extraction, which were executed in series. Figure 6 presents processing times, with Phi-4 being the fastest (2.95 s, IQR: 2.76–3.12), followed by Qwen2.5 (3.23 s, IQR: 3.04–3.38) and Gemma-2 (5.49 s, IQR: 5.27–6.06). Larger models, Llama 3.3 and DeepSeek-R1-Llama3.3, required longer times (9.45 s, IQR: 8.23–9.93 and 15.72 s, IQR: 14.87–16.72, respectively).

## 4. Discussion

Despite the benefits of structured reporting, adoption remains limited due to workflow challenges and clinician resistance. Addressing these issues could enhance radiology’s efficiency and data-driven capabilities. Recent LLM advancements offer automation opportunities [20,21,22,23], though many rely on closed-source models, raising concerns about patient confidentiality, regulatory compliance, and model oversight [20,21,22,24]. Jiang et al. [20] examined ChatGPT-3.5 and ChatGPT-4.0 for thyroid ultrasound reports, finding that while ChatGPT-3.5 produced satisfactory reports, ChatGPT-4.0 excelled in nodule categorization and management recommendations. Adams et al. [25] demonstrated GPT-4’s ability to convert 170 CT/MRI and 583 chest radiography reports into structured JSON files with 100% accuracy. Other studies explored the usability of open-weights LLMs to create structured reports [23,26,27]. Open-weight LLMs have also been explored for structured reporting. Woznicki et al. [26] assessed Llama-2-70B-chat for converting narrative chest radiograph reports into structured data, achieving human-comparable performance in English and German datasets. Other studies confirm that privacy-preserving open-weight LLMs can match closed models like GPT-4o in extracting structured data, underscoring their potential for automated clinical data processing [23].

Our study developed and validated an automated pipeline using open-weight LLMs and rule-based parsing to convert unstructured prostate MRI reports into structured data. A dataset of 250 reports was carefully curated to ensure diversity and minimize selection bias. The results demonstrated that models like DeepSeek-R1-Llama3.3, Llama3.3, and Phi-4 performed exceptionally well, though extraction accuracy varied for lesion-related data, indicating challenges in complex medical contexts. Variability in model performance across different physicians’ reports suggests that reporting style significantly impacts accuracy, highlighting the need for algorithm adaptation to specific physicians.

The automated approach provided substantial time and cost savings compared to manual reporting. Unlike radiologists, LLMs maintain consistent efficiency without fatigue and can process large volumes of reports daily. A hybrid workflow where AI generates structured reports from free text and radiologists review them could enhance efficiency while maintaining trust and accuracy. This study underscores the transformative potential of structured medical data, improving workflow efficiency, enhancing research, and unlocking valuable clinical insights.

This work has limitations. By centralizing the reference standard with a single radiologist, we aimed to minimize inconsistencies in interpretation while retaining a systematic methodology for extracting relevant clinical details. However, this strategy introduces the risk of subjective bias and precludes assessment of inter-rater reliability, which could offer insight into annotation consistency and model agreement with expert consensus.

Furthermore, all medical reports were sourced from a single institution and authored in Italian. This limits the model’s exposure to the variability in reporting styles that may exist across different clinical environments. Such stylistic differences, along with ambiguities in language and structure, may affect the generalizability and robustness of the model in broader settings. Future work should include datasets from multiple institutions, languages, and reporting styles to evaluate model performance under more diverse conditions.

## 5. Conclusions

This study demonstrates the strong capability of open-weight LLMs in extracting structured data from unstructured prostate MRI reports with high performance. All models, especially DeepSeek-R1-LLama3.3, Phi-4, and Llama3.3, exhibited exceptional performance across most features. The evaluation across different physicians’ reports revealed that reporting style significantly influences model performance, highlighting the need for prompt adaptation to individual radiologists’ styles to maximize performance. In terms of efficiency, automated extraction significantly reduced the time required for report generation. Compared to the manual process, LLMs operated with remarkable speed, achieving near-instantaneous results while maintaining high fidelity to the ground truth. Furthermore, incorporating an AI-assisted workflow, where radiologists review and modify AI-generated structured reports rather than compiling them from scratch, can be an option. This workflow aligns with real-world clinical practice by balancing automation with human oversight, ensuring reliable and interpretable outcomes.

Beyond workflow efficiency, this study underscores the broader potential of structured reporting in radiology. Converting free-text reports into structured formats unlocks vast amounts of valuable clinical data, facilitating improved patient care, research, and data-driven medical decision-making. Despite these advantages, challenges remain, particularly in ensuring robust generalization across diverse reporting styles and mitigating biases introduced by a centralized reference standard. Future work should explore adaptive prompt engineering and fine-tuning approaches to further optimize model performance across varied radiology practices.

Overall, our findings reinforce the viability of open-weight LLMs as powerful tools for structured reporting in radiology. These models can drive a transformative shift toward a more efficient, standardized, and data-driven radiology ecosystem.

## Figures and Tables

**Table 1:** Examples of free-text MRI reports and corresponding structured-output versions.

**ORIGINAL REPORT 1 (A)**					
Prostata di piccole dimensioni con relativa ipertrofia della zona transizionale. La prostata presenta diametri di **33 × 43 × 24 mm (cranio-caudale- latero laterale- antero-posteriore). Volume** complessivo di circa **18 cc**. **Densità del PSA 0.44 ng/mL^2^**. Relativa ipotrofia delle vescicole seminali con ristagno di secrezioni dense nel contesto. Capsula periferica prostatica continua. **Prostata transizionale**: ipertrofia stromale senza evidenza di noduli francamente ipointensi nella sequenza T2 pesata o restrizioni della diffusione. **PI-RADS 2**. **Prostata periferica**: presenza di aree di riduzione del segnale nella sequenza T2-pesata a livello di entrambi i lobi prostatici con presenza di nodulo di circa 1 cm di diametro al passaggio tra prostata sovra apicale e media postero-laterale sinistra caratterizzato da significativa riduzione del segnale nella mappa ADC (0.4 × 10^−3^ mm^2^/s) e significativa restrizione della diffusione. Curva di impregnazione con rapido wash-in. Il nodulo è prospiciente la capsula prostatica che non risulta superata. **PI-RADS 4**.					
**STRUCTURED REPORT 1**					
**Longitudinal**	**Transverse**	**Antero-post**	**Unit of meas.**	**Volume**	**PSA density**
33	43	24	mm	18	0.44
**Lesions**					
**PI-RADS**			**Location**		
2			Transition zone		
4			Peripheral zone		

**Table 2:** Examples of free-text MRI reports and corresponding structured-output versions. Values which are not indicated remain empty (-) in the structured report.

**ORIGINAL REPORT 2 (B)**					
DIMENSIONI Prostata ingrandita (diametri longitudinale **42 mm**, trasverso **52 mm**, antero-posteriore **36 mm**), per un volume complessivo di circa **41** cc. ASPETTI MORFOLOGICI Disomogeneita’ strutturale della regione centrale come per condizione di ipertrofia benigna, con adenoma del diametro longitudinale di 35 mm. Strie di basso segnale nelle immagini T2 a carico della porzione periferica, prevalentemente sinistra in verosimili esiti flogistici. LESIONI La valutazione della zona periferica soprattutto sinistra risulta limitata per la presenza di macroscopici artefatti da protesi d’anca sinistra. In particolare non è valutabile la pesature in diffusione. In sede **periferica** postero-laterale sinistra si rileva alterazione focale di 7 mm ipointensa in condizioni di base, apparentemente priva di enhancement contrastografico, compatibile con esito fibrotico. A causa dei limiti suddetti **non è possibile** a segnale una valutazione **PI-RADS** CAPSULA Conservato il profilo capsulare. VESCICOLE SEMINALI Nei limiti la morfologia delle vescicole seminali. VIE URINARIE Vescica distesa, con pareti lievemente ispessite, compatibile con vescica da sforzo. LINFONODI PELVICI Non sono evidenti adenopatie in sede iliaca e otturatoria.					
**STRUCTURED REPORT 2**					
**Longitudinal**	**Transverse**	**Antero-post**	**Unit of meas.**	**Volume**	**PSA density**
42	52	36	mm	41	-
**Lesion**					
**PI-RADS**			**Location**		
-			Peripheral zone		

**Table 3:** Each LLM includes special tokens unique to the model, which it uses to mark the beginning and end of structured components in its generation. These tokens help indicate the start or conclusion of a sequence, message, or response.

Model Name	Prompt
Llama3.3	*<|begin_of_text|><|start_header_id|>system<|end_header_id|>* ***{system_prompt}*** *<|eot_id|><|start_header_id|>user<|end_header_id|>* ***{user_prompt}*** *<|eot_id|><|start_header_id|>assistant<|end_header_id|>*
DeepSeek-R1-Llama3.3	<|begin▁of▁sentence|> **{system_prompt}** <|User|> **{user_prompt}** <|Assistant|>
Phi4	<|im_start|>system<|im_sep|> **{system_prompt}** <|im_end|><|im_start|>user<|im_sep|> **{user_prompt}** <|im_start|>assistant<|im_sep|>
Gemma-2	<start_of_turn>model **{system_prompt}** <end_of_turn><start_of_turn>user **{user_prompt}** <end_of_turn><start_of_turn>model
Qwen2.5-14B	<|im_start|>system **{system_prompt}** <|im_end|><|im_start|>user **{user_prompt}** <|im_end|><|im_start|>assistant

**Table 4:** Example illustrating the methodology used to compute the lesion score, detailing the individual parameters, their respective weightings, and the final score calculation.

LESION SCORE COMPUTATION				
**Initialize prediction list and ground truth list** ________________________________________________________________________________ Prediction list: [(PI-RADS: 4, Location: Transition Zone), (PI-RADS: 5, Location: Transition Zone), (PI-RADS: 2, Location: Peripheral Zone), (PI-RADS: 5, Location: Peripheral Zone)] Ground truth list: [(PI-RADS: 5, Location: Transition Zone), (PI-RADS: 4, Location: Transition Zone), (PI-RADS: 2, Location: Transition Zone)]				
**Create a blank schema array of the same length as the ground truth list** ________________________________________________________________________________ Blank Schema: [(), (), ()]				
**Loop over each lesion in the ground truth list:** If a complete match (same PI-RADS and Location) is found in the prediction list: Update the corresponding position in the blank schema. Remove the matched lesion from the prediction list. ________________________________________________________________________________ Example: Checking (PI-RADS: 5, Location: Transition Zone): Match found. Updated Blank Schema: [(PI-RADS: 5, Location: Transition Zone), (), ()] Remaining Predictions: [(PI-RADS: 4, Location: Transition Zone), (PI-RADS: 2, Location: Peripheral Zone), (PI-RADS: 5, Location: Peripheral Zone)] _ _ _ _ _ _ _ _ _ _ _ _ _ _ _ _ _ _ _ _ _ _ _ _ _ _ _ _ _ _ _ _ _ _ _ _ _ _ _ _ _ _ _ _ _ _ _ _ _ _ _ _ _ Checking (PI-RADS: 4, Location: Transition Zone): Match found. Updated Blank Schema: [(PI-RADS: 5, Location: Transition Zone), (PI-RADS: 4, Location: Transition Zone), ()] Remaining Predictions: [(PI-RADS: 2, Location: Peripheral Zone), (PI-RADS: 5, Location: Peripheral Zone)] _ _ _ _ _ _ _ _ _ _ _ _ _ _ _ _ _ _ _ _ _ _ _ _ _ _ _ _ _ _ _ _ _ _ _ _ _ _ _ _ _ _ _ _ _ _ _ _ _ _ _ _ _ Checking (PI-RADS: 2, Location: Transition Zone): Match **NOT** found. Blank Schema: [(PI-RADS: 5, Location: Transition Zone), (PI-RADS: 4, Location: Transition Zone), ()] Remaining Predictions: [(PI-RADS: 2, Location: Peripheral Zone), (PI-RADS: 5, Location: Peripheral Zone)]				
**Loop over remaining unmatched ground truth lesions:** If a partial match (same PI-RADS but different Location) is found in the prediction list: Update the corresponding position in the blank schema. Remove the matched lesion from the prediction list. ________________________________________________________________________________ Example: Checking for partial match: (PI-RADS: 2, Location: Peripheral Zone) found. Updated Blank Schema: [(PI-RADS: 5, Location: Transition Zone), (PI-RADS: 4, Location: Transition Zone), (PI-RADS: 2, Location: Peripheral Zone)] Remaining Predictions: [(PI-RADS: 5, Location: Peripheral Zone)]				
**Remaining lesions in the prediction list are considered false positives (FPs).** ________________________________________________________________________________ Example: Remaining Predictions: [(PI-RADS: 5, Location: Peripheral Zone)] False Positive: (PI-RADS: 5, Location: Peripheral Zone)				
**Output:** Prediction list: [(PI-RADS: 5, Location: Transition Zone), (PI-RADS: 4, Location: Transition Zone), (PI-RADS: 2, Location: Peripheral Zone), (PI-RADS: 5, Location: Peripheral Zone)]				
**F1 score evaluation:**				
Prediction		Evaluation	Ground truth	
**PI-RADS**	**LOCATION**		**PI-RADS**	**LOCATION**
**5**	**Transition zone**	TP, TP	**5**	**Transition zone**
**4**	**Transition zone**	TP, TP	**4**	**Transition zone**
**2**	**Peripheral zone**	TP, (FN + FP)	**2**	**Transition zone**
**5**	**Peripheral zone**	FP, FP	-	-
$F1=\frac{2\;*\;TP}{2\;*\;TP+FN+FP}$$=\frac{2\;*\;2.5}{2\;*\;2.5+0.5+1.5}$ = 0.71				

**Table 5:** (**A**). Summary of prostate characteristics, including dimensional measurements (longitudinal, transverse, and antero-posterior), volume, and PSA density, along with missing data counts. (**B**). Distribution of lesions based on PI-RADS score and anatomical location, providing an overview of lesion classification across different prostate zones.

STATISTICS SUMMARY (A)		
**Feature**	**Overall**	**Missing Data**
Age (years), mean ± SD	65 ± 9	0
Longitudinal dim (mm), median (IQR)	50 (43–57.5)	71
Transverse dim (mm), median (IQR)	53 (48–58)	71
Antero-posterior dim (mm), median (IQR)	39 (34–45)	71
Volume (cc), median (IQR)	51.5 (37.5–73.5)	36
PSA density (ng/mL^2^), median (IQR)	0.09 (0.06–0.14)	152
**LESIONS (B)**		
**PI-RADS Score**	**Location**	**Count (% of the total)**
PI-RADS 2	Peripheral Zone	136 (37.67%)
	Transition Zone	98 (27.14%)
	Peripheral and transition zone	2 (0.56%)
	Not specified	1 (0.27%)
PI-RADS 3	Peripheral Zone	31 (8.59%)
	Transition Zone	22 (6.1%)
	Peripheral and transition zone	0 (0%)
PI-RADS 4	Peripheral Zone	46 (12.74%)
	Transition Zone	11 (3.05%)
	Peripheral and transition zone	1 (0.27%)
PI-RADS 5	Peripheral Zone	9 (2.49%)
	Transition Zone	2 (0.56%)
	Peripheral and transition zone	2 (0.56%)

**Table 6:** Example of output obtained from each task execution.

Task	Expected Output
Dimension extraction	dimensions = [33, 43, 24] dimensionsOrder: [CC, LL, AP] measurement_unit: mm
Volume and PSA density extraction:	volume: 18 psa_density: 0.44
Lesion extraction	lesions: [(PI-RADS: 2, location: Transition zone), (PI-RADS: 4, location: Peripheral zone)]
Final aggregation	Exam: Dimensions: [Longitudinal: 33, Transverse: 43, Antero-posterior: 24, Unit of meas: mm] PSA density: 0.44 Volume: 18 Lesions: [(PI-RADS: 2, Transition zone), (PI-RADS: 4, Peripheral zone)]

**Table 7:** (**A**). Performances obtained by each model for each extracted feature. In bold, the best performance was highlighted. (**B**). Performance scores of each model, computed with respect to the physician (A–G). In bold, the best performance was highlighted.

FEATURE LEVEL (A)						
	Models					
Feature	Llama3.3	DeepSeek-R1	Phi4	Gemma2	Qwen2.5	Average ± SD (%)
Longitudinal dim	98.8%	**100%**	98.8%	92.8%	98.4%	97.8 ± 2.8
Transverse dim	99.2%	**100%**	98.8%	92.8%	99.6%	98.1 ± 3.0
Antero-posterior dim	98.8%	**100%**	98.8%	92.8%	98.8%	97.8 ± 2.9
Measurement unit	98.8%	99.2%	99.2%	**99.6%**	98.8%	99.1 ± 0.3
Volume	**100%**	98.4%	**100%**	**100%**	**100%**	99.7 ± 0.7
PSA density	**100%**	**100%**	**100%**	**100%**	**100%**	100 ± 0.0
Lesion count	90.8%	**94%**	93.6%	93.6%	88.4%	92.1 ± 2.4
Lesion score	**97.6%**	97.3%	95.8%	96.7%	95.7%	96.6 ± 0.9
Average ± SD (%)	98.0 ± 3.0	**98.6 ± 2.1**	98.1 ± 2.2	96.0 ± 3.4	97.5 ± 3.9	
**PHYSICIAN LEVEL (B)**						
	**Models**					
**Physician**	**Llama3.3**	**DeepSeek-R1**	**Phi4**	**Gemma2**	**Qwen2.5**	**Average ± SD (%)**
A	99.5%	99%	98.8%	98.6%	98.8%	98.9 ± 0.3
B	100%	100%	99.6%	100%	100%	**99.9 ± 0.2**
C	95.2%	96.9%	93.5%	90.9%	95.5%	94.4 ± 2.3
D	100%	98.1%	99.7%	98.9%	99.7%	99.3 ± 0.8
E	95.4%	97.9%	98.1%	97.9%	93.9%	96.6 ± 1.9
F	96.4%	100%	95%	85.5%	97.6%	94.9 ± 5.6
G	98.7%	98.3%	98.7%	98.8%	97.4%	98.4 ± 0.6
Average ± SD (%)	97.9 ± 2.2	**98.6 ± 1.1**	97.6 ± 2.4	95.8 ± 5.5	97.6 ± 2.2	

## Data Availability

The data presented in this study are available on request from the corresponding author.

## Abbreviations

The following abbreviations are used in this manuscript:

MRI	Magnetic Resonance Imaging
LLM	Large Language Models
IQR	Interquartile Range
SD	Standard Deviation
